# Applications of artificial intelligence in regenerative dentistry: promoting stem cell therapy and the scaffold development

**DOI:** 10.3389/fcell.2024.1497457

**Published:** 2024-12-06

**Authors:** Elham Saberian, Andrej Jenča, Andrej Jenča, Hadi Zare-Zardini, Mohammad Araghi, Adriána Petrášová, Janka Jenčová

**Affiliations:** ^1^ Klinika of Stomatology and Maxillofacial Surgery Akadémia Košice Bacikova, Pavol Jozef Šafárik University, Kosice, Slovakia; ^2^ Klinika of Stomatology and Maxillofacial Surgery Akadémia Košice Bacikova, UPJS LF, Kosice, Slovakia; ^3^ Department of Biomedical Engineering, Meybod University, Meybod, Iran; ^4^ Department of Computer Engineering, The University of Tehran, Tehran, Iran; ^5^ Klinika of Stomatology and Maxillofacial Surgery Akadémia Košice Bacikova, Pavol Jozef Safarik University, Kosice, Slovakia

**Keywords:** dental, artificial intelligence, regenerative medicine, tooth, stem cell (SC)

## Abstract

Tissue repair represents a critical concern within the domain of dentistry. On a daily basis, countless individuals seek dental clinic services due to inadequate dental care. Many of the treatments that patients receive have unfavorable side effects. The employment of innovative methodologies, including gene therapy, tissue engineering, and stem cell (SCs) applications for regenerative purposes, has garnered significant interest over the past years. In recent times, artificial intelligence, particularly neural networks, has emerged as a topic of considerable attention among many medical professionals. Artificial intelligence possesses the capability to analyze data patterns through learning algorithms. Research opportunities in the rapidly expanding field of health sciences have been made possible by the use of artificial intelligence (AI) technologies. Though its uses are not restricted to these situations, artificial intelligence (AI) has the potential to improve and accelerate many aspects of regenerative medicine research and development, especially when working with complicated patterns. This review article is to investigate how artificial intelligence might be used to enhance regenerative processes in dentistry by using scaffolds and stem cells, in light of the continuous advances in artificial intelligence in the fields of medicine and tissue regeneration. It highlights the difficulties that still exist in this developing sector and explores the possible uses of AI with a particular emphasis on dentistry practices.

## 1 Introduction

Recently, AI has grown in a number of ways thanks to developments in needs of computer hardware, software, and algorithms. Artificial intelligence is a field of computer science that lets machines work and solve problems in the same way as people do. Due to the feasibility of mimicking cognitive functions and tidning error experiences for performance enhancement; this technology has risen in popularity in the medical and dental sectors (Bays et al., 2023; Nelson et al., 2020). Advancements in other subsections of AI like machine learning (ML), deep learning (DL), and natural language processing (NLP) have enhanced the possible uses of AI in delivering efficient diagnostic, therapeutic and regenerative medicine solutions (Lauriola et al., 2022). ML being a subcategory of AI uses coded data and comes up with improved patterns out of the results it produces by learning from the mistakes it makes through iterative changes (Alam, 2021; Kaul et al., 2020). A subcategory of ML, deep learning applies artificial neural networks based on human cortical ones, including their connections and functions as carries and processors of information (Liu et al., 2020; Qayyum et al., 2020). DL uses artificial neural networks, which are based on the structure of neurons, and data processing occurs in layers, working with data in numeric form to conduct analyses on large and various datasets. This makes it possible for DL algorithms to detect patterns, which might be intangible to humans from differences in the images (Asmika et al., 2021; Sakshi et al., 2022). Furthermore, DL algorithms have short term memory and long term memory which makes them capable of adjusting dynamically as well as producing better results with incoming data feeding (Mandalapu et al., 2023). Such features have placed AI in different domains like biomedical engineering, image identification, and tissue engineering (Taye, 2023; Wan et al., 2021). Certainly, in the field of dentistry AI adoption is promising, especially when implemented in regenerative medicine. The most researched and likely to succeed comes under the category of bioengineering of dental tissues where stem cell along with scaffold are used for replacing the damaged or necrotic tissue. For example, dental pulp stem cells which can be sourced from the dental pulp complex can be effectively used to repair dental pulp tissue after it has been destroyed (Hongen et al., 2021). AI can help to enhance these processes with the help of the analysis of factors that define cells’ differentiation, creating an optimal scaffold for cell growth, and predicting the effectiveness of the treatment. In endodontics, AI has shown an efficacy in predicting the shape of the root canal, the presence of periapical lesions, and the viability of the dental pulp stem cells, as well as the general prognosis of the applied methods of treatment (Yang et al., 2021; Nosrati and Nosrati, 2023). AI also holds the possibility of improving diagnostic functions and organization of the approaches to be used in root canal treatment. However, the present uses of AI in regenerative dentistry are still limited and can be considered in their embryonic form and are in need for further investigation in order to elucidate their potential, opportunities, threats and barriers (Tsutsui, 2020; Agrawal and Nikhade, 2022). The review aims at presenting key and innovative implementations of Artificial Intelligence in the field of stem cell and scaffold based regenerative medicines and dentistry. More precisely in this paper, the ways AI can be used to improve stem cell differentiation, enhance scaffold structures as well as give prediction of the treatment results, and foster regenerative endodontics will be discussed. Thus, through a metanalytical approach to the literature in the domain, this review seeks to establish an understanding of current and potential future issues and directions for AI work in regenerative therapeutics. To this end, a search was done on databases like PubMed, Google Scholar and Web of Science using the keywords: artificial intelligence, dentistry, stem cells, scaffolds, regeneration, deep learning and machine learning.

## 2 Methodology

This review was performed with the use of the narrative approach, as the aim of the present review was to identify and discuss the current literature on the use of artificial intelligence in the field of regenerative dentistry, with the focus on stem cells and scaffolds. Unlike the systematic reviews, the current narrative review did not use specific inclusion or exclusion criteria. On the other hand, any article that could be related to the topic from the title, abstract, or content was included to ensure that the study offered an overview of the field. The literature search was performed using three major databases: The related studies were searched from the PubMed, Google Scholar, and Web of Science databases. The search was conducted using a combination of the following keywords: AI, Dentistry, Regeneration, Stem cells, Scaffolds, Dental cell therapy, Deep learning and Machine learning. The AND and OR operators were used in the search process to connect the keywords. For example, the following queries were used: artificial intelligence dentistry regeneration, machine learning deep learning stem cells scaffolds. The search was done up to November 2023, for articles only.

There were no strict criteria for the inclusion or exclusion of studies as the present study was a narrative review which set out to map the literature in this interdisciplinary research area. Only articles that contained information about AI applications in regeneration of dental tissues were considered and included in the study. These included:

1. Original Research Articles: Papers that explored AI in stem cell therapy, scaffold, or regenerative treatment in dentistry.
2. Review Articles: Systematic reviews analyzing AI in regenerative medicine, or its use in the field of dentistry.
3. Theoretical and Experimental Studies: Papers that either suggest or compare AI solutions to dental regeneration or associated areas.

Full-text articles were screened and selected based on their relevance to the topic and ability to determine key ideas, developments and issues regarding the application of AI for regenerative dentistry.

## 3 AI applications in dentistry

In recent years, dentistry has been changed by ML and has offered new methods to advance diagnostics, treatment, and regenerative medicine. Unlike traditional methods, AI employs substantial amounts of data and complex computations to process the data, identify the pattern, and make decisions that, in the past, could be made only with knowledge and experience. In particular, in the recent years developed AI technologies such as machine learning (ML), deep learning (DL), convolutional neural networks (CNNs) and others have made numerous applications in dentistry more precise especially in diagnostics, imaging, treatment planning and regenerative medicine. The subsequent part of the paper focuses on the implications of AI in dentistry as well as an emphasis on different applications and emerging technologies (Lee et al., 2018a; Jaiswal and Bhirud, 2021; Zhou, 2017).

### 3.1 AI in dental imaging and diagnostics

The most common application of AI in dentistry is in imaging and diagnostics where the technology is perhaps the most famous. With increased use of AI based systems, diagnosis of dental pathologies such as caries, periodontal diseases and oral cancer has been found to be more effective. For instance, deep learning methods specifically Convolutional neural networks (CNNs) have been used in analyzing Dental radiograph, Panoramic X-rays, Cone Beam Computed Tomography (CBCT) scans.

Recent advancements include:

- Caries Detection: CNNs have even recorded higher accuracy in identifying early caries lesions on bitewing radiographs than the dentists. For instance, Lee et al., (2023) showed that AI model had a sensitivity of 92% for diagnosing early-stage caries than the human evaluators with 82% sensitivity.
- Periodontal Disease Diagnosis: CBCT scans have been used to analyze bone loss and periodontal health and AI has been used on these scans to do the same. The changes of alveolar bone levels can be effectively identified and measured using machine learning algorithms, which will be beneficial for clinicians in early diagnosis and treatment planning.
- Oral Cancer Detection: Machine learning algorithms specifically using histological and imaging information have been applied to accurately diagnose oral cancers. For example, a research done early this year determined that a deep learning algorithm had a 95% accuracy rate in distinguishing between malignant and benign oral lesions from biopsy images.

These developments also mean that there is early detection of the diseases causing teeth problems hence early treatment is enhanced and the health of the patients improved (LeCun et al., 2015; Nam, 2020).

### 3.2 AI in endodontics

AI has been adopted by endodontics to enhance the precision of the root canal treatment. Deep learning algorithms have been utilized for:

- Root Canal Anatomy Identification: AI can read CBCT images and accurately determine complicated root canal anatomies which are difficult to visually assess. This minimizes the chances of failing to locate the canals and thus enhances the treatment results.
- Periapical Lesion Detection: Consequently, AI systems are capable of detecting periapical lesions with high accuracy from radiographic images including situations where the lesions are diminutive or genuinely visualized by the naked eye. A study conducted in 2023 showed that a CNN based diagnosis had a 94% diagnostic accuracy in diagnosing periapical lesions that is higher than conventional diagnostic methods.
- Treatment Success Prediction: Computer models have been designed to forecast the probabilities of the outcomes of root canal treatments depending on radiographic findings, patients’ information, and treatment variables.

These developments help to minimize the possibilities of mistakes when diagnosing and treating patients on the endodontic therapies successfully (Leya et al., 2023; Chae et al., 2011; Wu et al., 2016).

### 3.3 AI in orthodontics

Another area where AI has made a massive difference is Orthodontics especially in diagnosis and treatment. Key applications include:

- Cephalometric Analysis: In cephalometric radiographs, AI algorithms can work on cephalometric landmark detection without much time and effort for manual tracing. A recent deep learning model achieved landmark detection accuracy greater than 90% which greatly enhanced the effectiveness of orthodontic treatment planning.
- Treatment Simulation: Invisalign’s ClinCheck is an example of how machine learning is incorporated in AI-based software to plan the treatment and show the results digitally.
- Growth Prediction: AI algorithms have been trained to predict the growth of the craniofacial structures in children so that the correct time for treatment can be determined.

These tools have improved the accuracy as well as reliability of the orthodontic procedures, and offers individualized treatment plans for every patient (LeCun et al., 2015; Chae et al., 2011).

### 3.4 The application of AI in prosthodontics and restorative dentistry

In the fields of prosthodontics and restorative dentistry, AI is applied to create and produce dental restorations more accurately and productively.

- Digital Smile Design (DSD): DSD systems with artificial intelligence generate patient photos and facial structure to develop individual smiles. It also enables the dentists to see the final look of the restoration and make communication with the patient easier.
- Crown and Bridge Design: CAD/CAM technologies connected to AI can create crowns, bridges, and inlays that require little adaptation and can be produced quickly at the chairside.
- Material Selection: AI models can forecast behavior of various restorative materials in certain circumstances and help in choosing materials for long term restorations.

The developments have brought about enhanced customer satisfaction and decreased operational losses in restorative dentistry (Chae et al., 2011; Wu et al., 2016; Yauney et al., 2019).

### 3.5 AI in regenerative dentistry

As already indicated, one of the most promising areas of AI application in dentistry is regenerative medicine. AI has been employed for determining stem cell based therapies and scaffold structures for dental tissue engineering.

- Stem Cell Differentiation: AI models can analyze gene expression data and predict the conditions for differentiation of mesenchymal stem cells (MSCs) into odontoblast like cells. For example, machine learning algorithm designed in 2022 discovered signaling pathways that increase the effectiveness of stem cell differentiation by 30%.
- Scaffold Design: Different generative designs are being applied to AI to generate scaffolds with targeted porosity, mechanical strength, and biocompatibility. Specifically, these scaffolds offer enhanced support to cell adhesion and growth to enhance tissue repair.
- Predictive Modeling: The use of AI in regenerative medicine is useful in predicting the effectiveness of the treatments depending on the patient, age, health conditions and genetic makeup of the person (Lee et al., 2018a; Jaiswal and Bhirud, 2021; Leya et al., 2023; Wu et al., 2016). This progress shows significant potential that future regenerative dentistry is more effective, individualized and successful.

## 4 Artificial intelligence and clinical solutions based on stem cells

Among the innovative applications of artificial intelligence is the modeling of dental research. Utilizing neural network algorithms, laboratory modeling of oral diseases has been explored, along through the investigation of prospective cures and the underlying causes of disease. Through disease modeling, artificial intelligence can furnish dentists with detailed insights into the pathology of diseases and identify novel treatment targets (Lee et al., 2018a; Jaiswal and Bhirud, 2021). Various innovations have been introduced concerning the integration of artificial intelligence with healthcare and dentistry (Zhou, 2017; LeCun et al., 2015). Moreover, artificial intelligence has been employed as a solution to enhance treatment processes and precision in clinical decision-making (LeCun et al., 2015; Nam, 2020).

**FIGURE 1:**
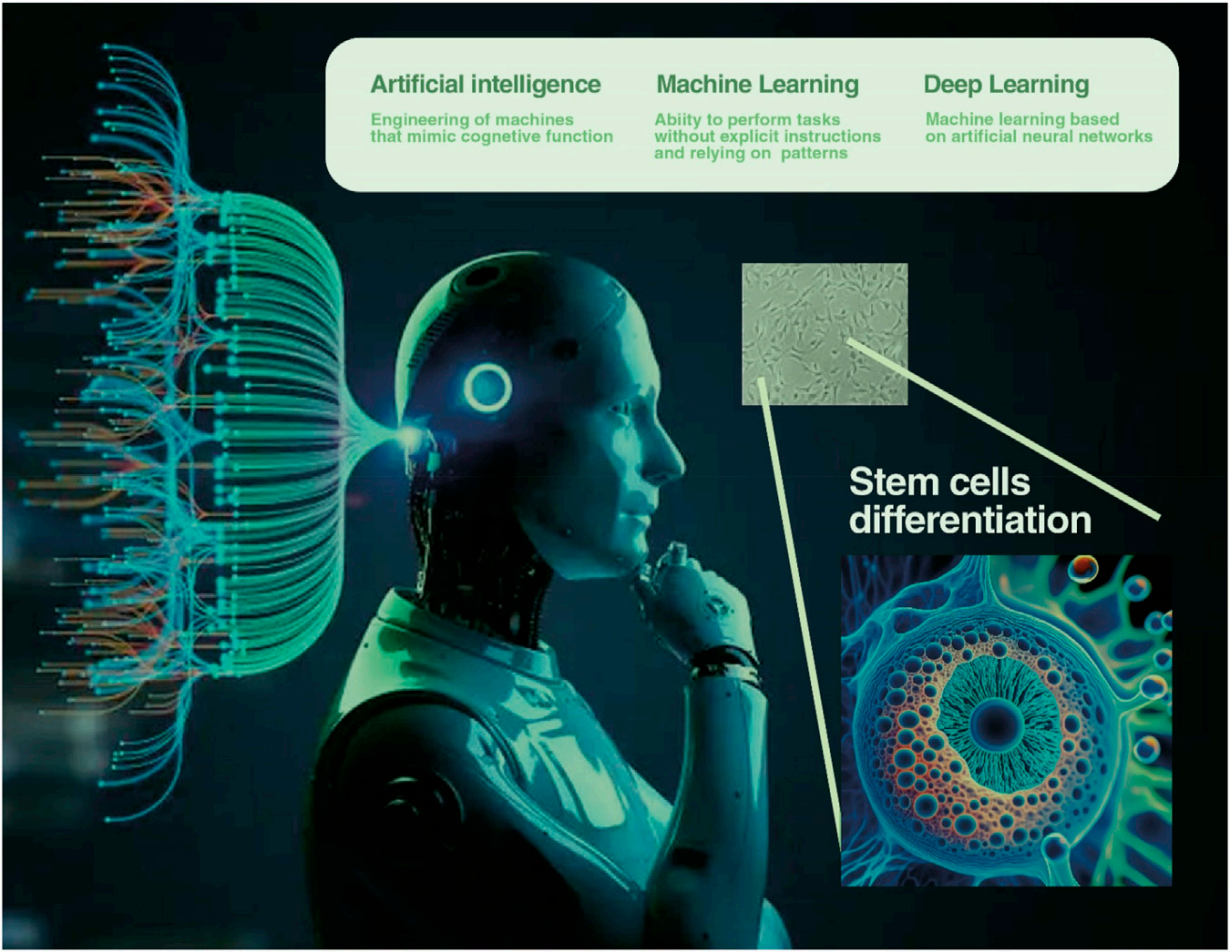
Artificial intelligence includes different subsets that can help to better identify the biological characteristics (differentiation, morphology of stem cells, etc.) of stem cells by identifying different algorithms. Deep learning-based algorithms image cells in an automated process, reducing the time-consuming process that is often done manually.

Stem cell-based therapies often suffer from poor efficacy due to cellular heterogeneity and inconsistency in therapeutic effectiveness, partly attributed to the senescence of mesenchymal stem cells (MSCs). In a study involving the modeling of mesenchymal stem cells (MSCs) with the aid of artificial intelligence algorithms and immunofluorescence imaging, markers of MSC aging—structures related to stem cell morphology—were identified (Figure 1). This study showed that at the stem cell level, the expression levels of age indicators, such as beta-galactosidase, were predictable. With this system, non-senescent and senescent mesenchymal stem cells can be differentiated, and the microscope-based phenotype platform can be integrated into routine cell culture practices, transforming it into a facile tool for MSC engineering and production (Leya et al., 2023). Mesenchymal stem cells (MSCs), with their ease of isolation, multipotent, immunocompetent, and immunosuppressive properties, hold promising potential in biomedical and dental research therapies. Enhancing our understanding of these cells through artificial intelligence could represent a significant step forward in the treatment of numerous diseases.

In the past, artificial intelligence (AI) tools have been employed in the prediction of drug and biological reaction outcomes. DeepChem, a Python package grounded in deep learning, is capable of forecasting molecular properties and screening prospective drugs. Other analogous tools, such as DeltaVina and AlphaFold, which are based on deep learning, are recognized for their ability to predict protein structure and their interactions with protein ligands (Chae et al., 2011; Wu et al., 2016). The applications of AI have been assessed across various domains of dentistry. In this review article, the focal point is on artificial intelligence and its role in augmenting the functionality of stem cells within the context of dentistry.

## 5 Applications of artificial intelligence in dentistry

**FIGURE 2:**
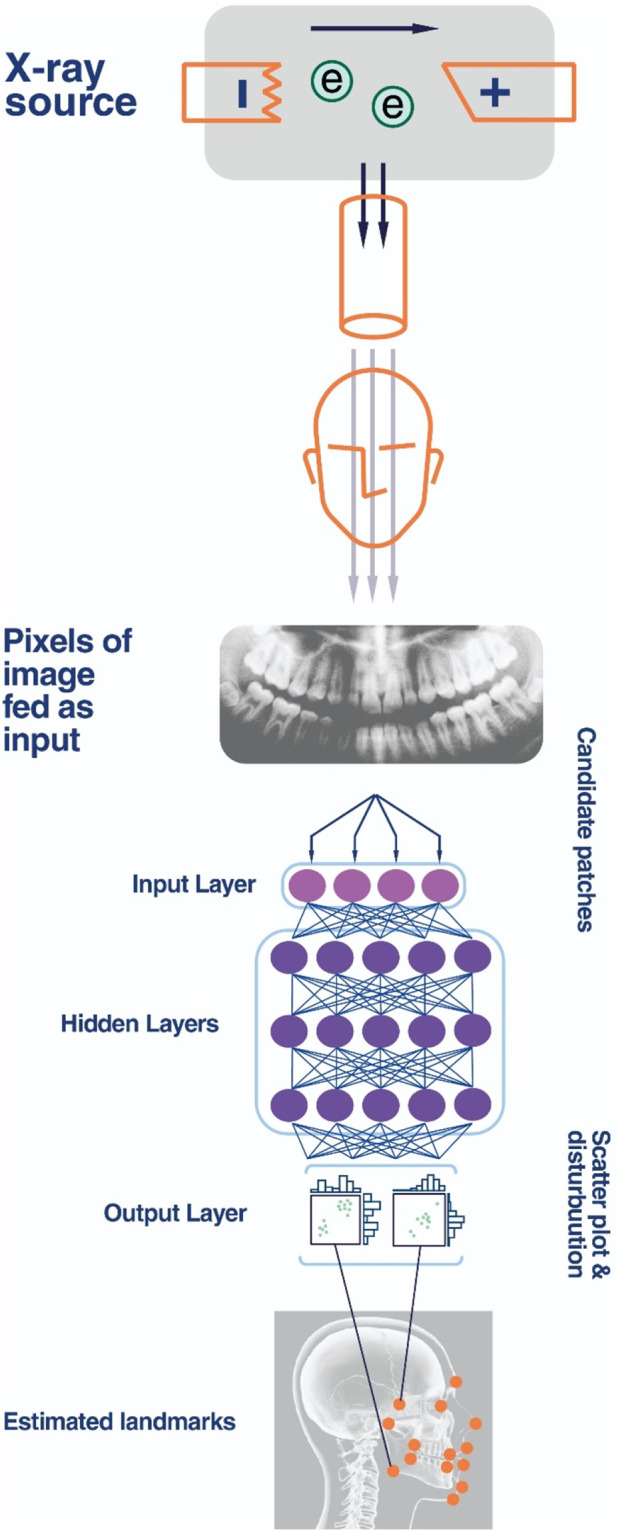
CNN for deep learning and dentistry.

Artificial intelligence (AI) applications have been utilized in the treatment of oral and dental diseases. As previously mentioned, AI applications leverage existing data for specialized algorithms. In dentistry, the desired data are typically encoded and assessed from panoramic and periapical radiograph findings, Cone Beam Computed Tomography (CBCT), and intraoral images. Early identification of tooth decay, particularly in the presence of secondary lesions, poses a diagnostic challenge. AI has proven beneficial in diagnosing tooth decay, vertical root fractures, apical lesions, volumetric evaluation of the pulp space, and assessment of tooth wear (Yauney et al., 2019; Huang and Lee, 2021; Fukuda et al., 2020; Vadlamani, 2020; Setzer et al., 2020). Additionally, studies indicate that AI is superior to dentists in the diagnosis of proximal caries (Schwendicke et al., 2021). One form of deep learning model that is commonly used is Convolutional Neural Networks (CNNs), which are mostly used for picture production and recognition (Figure 2). In one study, a CNN algorithm that used periapical radiographs to identify tooth decay had an accuracy and sensitivity of roughly 86% and 90%, respectively (Lee et al., 2018a). In another research, the CNN algorithm was applied to detect caries in intraoral images, with reported accuracy and sensitivity of around 92%–93% (Kühnisch et al., 2021). AI has yielded acceptable results in the diagnosis of periodontal diseases, with the CNN algorithm exhibiting lower sensitivity than in tooth decay detection but generally above 70% in most cases (Yauney et al., 2019; Krois et al., 2019; Kim et al., 2020; Huang et al., 2020; Lee et al., 2018b; Tanikawa et al., 2021). In orthodontics, AI has also shown positive outcomes (Tanikawa et al., 2021; Thanathornwong, 2018), contributing significantly to treatment effectiveness by simulating pre- and post-treatment images (Hwang et al., 2020; Wei et al., 2022a).

## 6 Dentistry can be more effective with the help of cell therapy and AI

The loss of teeth, jawbone, or both, as a consequence of periodontal disease, dental caries, trauma, or certain genetic disorders, impacts not only the fundamental functions of the oral cavity but also its aesthetics and the quality of life. Modern dentistry has addressed this issue through the application of tissue grafting techniques. The bioengineering of teeth with stem cells represents a promising avenue for the regeneration of individual teeth. Some studies suggest that following dental pulp necrosis, stem cells from the dental pulp complex can be harnessed to generate dental pulp, which, when placed within formed channels, closely resembles dental dentin (Dubuc et al., 2022).

In the course of various investigations, Observations have shown that cell therapy can play a regenerative role of significant efficacy in endodontic treatments (Yan et al., 2023; Cho et al., 2022). The exceptional characteristics of stem cells, in comparison with alternative treatment modalities and pharmaceuticals, have recently piqued the interest of dentists as a therapeutic tool. Stem cells possess two distinctive and defining features: the capacity to differentiate into various tissues and the ability for self-renewal, which have established them as an effective instrument in cell therapy. Living cells are used in cell therapy to replace or treat diseased or damaged tissues and organs (Tanikawa et al., 2021).

**FIGURE 3:**
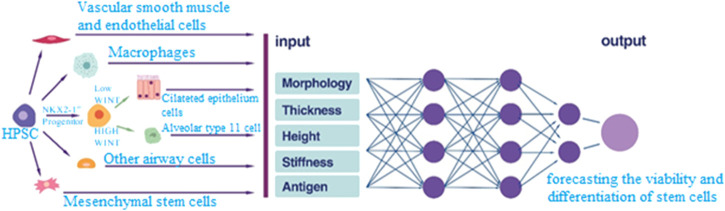
Different types of stem cells are analyzed using artificial intelligence’s assistance tools in terms of surface antigens, morphology, size and volume. The patterns are analyzed algorithmically and live and differentiated cells are identified.

At present, stem cell therapy is regarded as an appealing and progressive domain within medical science and disease treatment. Even if cell treatment has demonstrated encouraging results in a number of diseases, it continues to confront major obstacles in finding the right cells, guaranteeing patient safety, and maximizing these cells’ performance (Hwang et al., 2020; Wei et al., 2022b). Artificial intelligence (AI), with its capability to encode and algorithmically process data, can play a pivotal role in identifying appropriate cells and enhancing their effectiveness (Leya et al., 2023). AI is also adept at determining optimal conditions for cell growth. Artificial intelligence algorithms can determine which parts of the gums and teeth are most likely to become inflamed in the future by examining a medical history and genetic information about the patient (Wei et al., 2022b). The identification of stem cells with appropriate biological properties is crucial in regenerative dentistry. Indeed, based on the results obtained, AI proves beneficial in tissue engineering (Mackay et al., 2021) (Figure 3). Undoubtedly, artificial intelligence holds the potential to transform dentistry by identifying differentiated and high-quality cells.

Artificial intelligence (AI) can assess the factors that effectively enhance the differentiation and quality of differentiated oral cells. In one study, the neuro-fuzzy inference approach was used to assess the potential applications of dental pulp-derived stem cells in various restorative procedures. This method facilitated the prediction of stem cell survival following exposure to bacteria in a clinical model (Bindal et al., 2017). In another research, it was observed that after the injection of lipopolysaccharide into pulp stem cells to induce an inflammatory response, the viability of the cells was altered, and the accuracy of AI prediction in examining the survival of these stem cells post-microbial invasion was substantial. The researchers noted that artificial intelligence can evaluate cellular changes in real-time (Aminoshariae et al., 2021).

Beyond stem cells, the scaffold plays an indispensable function in dental stem cell applications. Findings indicate that the type of scaffold influences the distinction of human dental pulp stem cells and accelerates the pace of regeneration (Zhang et al., 2016). On the other hand, the efficacious role of artificial intelligence in tissue engineering scaffolds has been established (Bermejillo Barrera et al., 2021). Potentially, with the aid of artificial intelligence, it is feasible to predict the optimal structure for the scaffold, which is of significant importance in dentistry and regeneration. A large amount of study has been done on the relationship between artificial intelligence and oral diseases (Vadlamani, 2020; Kühnisch et al., 2021). However, given the paucity of studies in the domain of regeneration involving dental stem cells, conducting novel studies in this area could contribute to a deeper understanding of this tool’s application in tooth tissue regeneration.

## 7 Artificial intelligence and stem cells

Treatment with stem cells (SCs) has demonstrated efficacy across a broad spectrum of diseases. Machine learning and deep learning, two pivotal domains of artificial intelligence, are anticipated to play an indispensable function in the future of global healthcare through their applications in stem cell research (Shende and Devlekar, 2021). Through predictive modeling, artificial intelligence models built on machine learning algorithms can accelerate the development and formulation of cell treatments. This technology can select the most suitable stem cells based on their biological attributes for patient treatment. Researchers have posited that artificial intelligence may prove instrumental in classifying stem cell colonies, distinguishing cell morphology, and differentiating between healthy and unhealthy cells, thereby enhancing diagnostic accuracy (Shende and Devlekar, 2021; Srinivasan et al., 2021).

**FIGURE 4:**
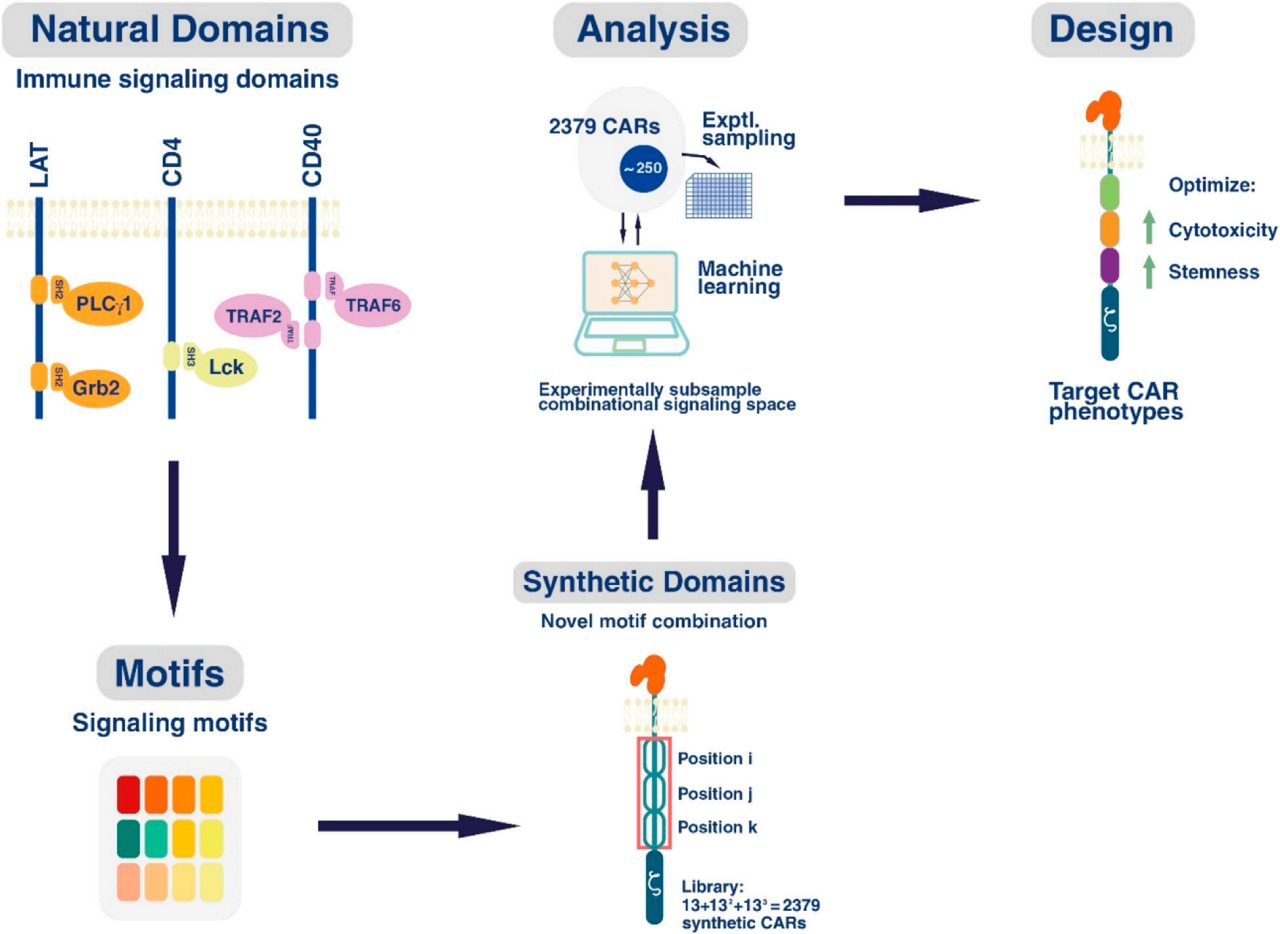
Artificial intelligence with the help of machine learning can improve the efficiency of cytotoxicity with new engineering.

A study revealed that machine learning could be harnessed to uncover fundamental design principles for the creation of a suitable microenvironment conducive to stem cell development, thereby averting cellular abnormalities (Zaman et al., 2021). Chimeric Antigen Receptor (CAR) T-cell therapy was accepted as the inaugural drug product for the treatment of acute leukemia and lymphoma (EMA/188757/2022 Kymriah, 2022). The decoding of CAR T-cell phenotype utilizing combinatorial signaling motif libraries and machine learning was explored in a research study. This investigation examined signaling motifs that interface with CAR T-cell receptors and assessed the impact of hundreds of motifs with varying signaling properties on CAR T-cell tumor killing, leveraging the capabilities of artificial intelligence. In this design, machine learning algorithms were trained on genetic data to forecast CAR T-cell phenotype encoded by signaling motif combinations (Daniels et al., 2022) (Figure 4). The results of the investigation indicated that the novel rules derived from this modeling could influence the enhanced engineering of stem cells and tissue regeneration (Naghizadeh et al., 2022).

The effectiveness of artificial intelligence (AI) in enhancing the quality of stem cell manufacturing and delivery has been well-established. AI aids in assessing the viability, efficacy, effectiveness, and safety of stem cells. Also, in the context of regenerative medicine involving stem cells, AI simplifies simulation and modeling processes while elucidating the relationship between the functions of cells and their surroundings (Capponi and Daniels, 2023; Umar, 2023). Deep learning models in AI have proven effective in distinguishing between various cell morphologies. Using AI tools, features such as colony characteristics, classification, differentiation, and morphological traits of hematopoietic stem cells have been evaluated (Shouval et al., 2015; Fan et al., 2017; Kavitha et al., 2017; Waisman et al., 2019; Schaub et al., 2020; Aida et al., 2020; Juhola et al., 2021). Additionally, AI has been employed in predictive studies of mesenchymal cell differentiation. Using publicly available RNA-seq data on the tri-lineage differentiation of human mesenchymal stem cells (hMSCs), this review creates a reference gene expression profile distinct to each cell type. Additionally, a prediction model utilizing the k-nearest neighbors (kNN) method is presented, which categorizes hMSC differentiation lineages (Zhou et al., 2023). Table 1 summarizes studies that have utilized AI in predicting and identifying the risk of leukemia, as well as analyzing stem cell morphology.

**TABLE 1:** Data from studies conducted on stem cells with the help of artificial intelligence.

Study	Type of study	Study type of stem cell	Findings/Conclusion
Shouval et al. (2015)	Retrospective cohort	Allogeneic hematopoietic stem-cel	The data mining method proved to help predict 100-day overall mortality and has extended that prediction to up to 2 years The data mining method proved to help predict 100-day overall mortality and has extended that prediction to up to 2 years The data mining method proved to help predict 100-day overall mortality and has extended that prediction to up to 2 years The data mining method proved to help predict 100-day overall mortality and has extended that prediction to up to 2 years. This tool is available online to aid in the risk evaluation of patients with acute leukemia before hematopoietic stem cell therapy The data mining method proved to help predict 100-day overall mortality and has extended that prediction to up to 2 years. This tool is available online to aid in the risk evaluation of patients with acute leukemia before hematopoietic stem cell therapy بیب The data mining method proved to help predict 100-day overall mortality and has extended that prediction to up to 2 years. This tool is available online to aid in the risk evaluation of patients with acute leukemia before hematopoietic stem cell therapy The data mining method proved to help predict 100-day overall mortality and has extended that prediction to up to 2 years. This tool is available online to aid in the risk evaluation of patients with acute leukemia before hematopoietic stem cell therapy The data mining method proved to help predict 100-day overall mortality and has extended that prediction to up to 2 years. This tool is available online to aid in the risk evaluation of patients with acute leukemia before hematopoietic stem cell therapy The data mining method proved to help predict 100-day overall mortality and has extended that prediction to up to 2 years. This tool is available online to aid in the risk evaluation of patients with acute leukemia before hematopoietic stem cell therapy n day 100, the prevalence of overall mortality (OM) was 13.9%. The model analyzed ten variables, and the crude score was adjusted for 100-day OM according to individual probabilities using a logistic transformation method (range 3%–68%). The primary outcome determined by the model outperformed the European Group for Blood and Marrow Transplantation score (area under the receiver operating characteristic curve, 0.701 vs. 0.646; *p* = 0.001). The calibration was considered flawless, and the secondary objectives were also predicted according to the allocated scores n day 100, the prevalence of overall mortality (OM) was 13.9%. The model analyzed ten variables, and the crude score was adjusted for 100-day OM according to individual probabilities using a logistic transformation method (range 3%–68%). The primary outcome determined by the model outperformed the European Group for Blood and Marrow Transplantation score (area under the receiver operating characteristic curve, 0.701 vs. 0.646; *p* = 0.001). The calibration was considered flawless, and the secondary objectives were also predicted according to the allocated scores n day 100, the prevalence of overall mortality (OM) was 13.9%. The model analyzed ten variables, and the crude score was adjusted for 100-day OM according to individual probabilities using a logistic transformation method (range 3%–68%). The primary outcome determined by the model outperformed the European Group for Blood and Marrow Transplantation score (area under the receiver operating characteristic curve, 0.701 vs. 0.646; *p* = 0.001). The calibration was considered flawless, and the secondary objectives were also predicted according to the allocated scores The data mining method proved to help predict 100-day overall mortality and has extended that prediction to up to 2 years. This tool is available online to aid in the risk evaluation of patients with acute leukemia before hematopoietic stem cell therapy The data mining method proved to help predict 100-day overall mortality and has extended that prediction to up to 2 years. This tool is available online to aid in the risk evaluation of patients with acute leukemia before hematopoietic stem cell therapy Prior to receiving hematopoietic stem cell treatment, this tool assists in determining the risk of acute leukemia patients
Fan et al. (2017)	Quasi-experiment	iPSC	The artificial intelligence tool detected and predicted the high colony feature very well
Kavitha et al. (2017)	Animal study	iPSC	A trustworthy framework for classifying iPSC colonies is V-CNN.
Waisman et al. (2019)	Quasi-experiment	Mouse embryonic stem cells	The network was effective in identifying undifferentiated and differentiated cells with an accuracy of more than 99% and was able to distinguish cell morphology well
Schaub et al. (2020)	Quasi-experiment	iPSC-RPE	Machine learning makes it feasible to characterize noninvasive cell treatment
Aida et al. (2020)	Animal study	Cancer stem cells	Artificial intelligence using fluorescence images is effective in identifying morphological traits of cells
Juhola et al. (2021)	Quasi-experiment	iPSC Cardiomyocytes	The effects of iPSC-CM medicine may be analyzed using machine learning techniques
Zhou et al. (2023)	Quasi-experiment	hMSCs	Predicting the lineage fate of stem cells and assessing the initial functional properties of biomaterials can be effectively and precisely done with machine learning
Katarzyna et al. (2023)	Quasi-experiment	hPSC-HEP	Deep learning models provide an alternate method for functionally characterizing stem cell cultures since they are able to differentiate between various cell morphologies

iPSC, induced pluripotent stem cells; V-CNN, vector-based convolutional neural network; iPSC-RPE, induced pluripotent stem cells-retinal pigment epithelial, hPSC-HEP, Human mesenchymal stem cells, or hMSCs, are the source of human pluripotent stem cell-derived hepatocytes.

## 8 Discussion

Artificial intelligence (AI) is increasingly utilized in medical research through computational simulations and *in silico* studies. Its quicker outcomes and less expensive than clinical and laboratory methods have established AI as a valuable and promising approach (Salaha et al., 2023; Tauviqirrahman et al., 2023; usheel Kumar et al., 2023). These initiatives seek to use artificial intelligence (AI) to enhance and expedite a range of procedures, including medication development and dental care. Researchers and dentists hope to improve the people’s quality of life and societies in the long run by integrating AI to produce more precise and efficient results (Smojver et al., 2022; Wang et al., 2024).

Artificial Intelligence has the capability to efficiently detect biomarkers, genetic structures, and additional elements that support the regeneration of dental tissue. Additionally, this information can be used to develop gene- or cell-based treatments that are customized for each patient. Identifying highly efficient cells using AI will undoubtedly impact the regeneration process, thereby enhancing the quality of oral and dental treatment.

Differentiation is a crucial factor in stem cell culture, making it essential to identify differentiated cells within cultured cell mixtures. According to the studies we reviewed, AI can significantly contribute to identifying these cells and assessing cell differentiation. AI is resistant to training data errors and has been successfully applied to challenges such as speech recognition, image analysis and interpretation, and robotic learning. As previously noted, AI’s positive performance in scaffold design has been demonstrated (Bermejillo Barrera et al., 2021). Thus, utilizing AI in scaffold design and identifying suitable stem cells is likely to enhance regenerative outcomes.

The human brain comprises roughly 10^11^ neurons, each connected to around 10^4^ other neurons. The switching speed of neurons is about 10^–3^ s, considerably slower than computers (10^–10^ s). In general, AI enables computers to think, reason, and solve problems similarly to humans. AI can analyze and synthesize large volumes of data in a very short period, much faster than humans. By considering all dental data related to stem cell regeneration and quality, more efficient and rational decisions can be made.

With a lot of research being done on understanding stem cell behavior, identifying stem cells, and distinguishing differentiation kinds, artificial intelligence (AI) is one of the burgeoning topics in science and computer engineering (Shende and Devlekar, 2021). However, there remains a significant need for further research and the development of new algorithms in the field of regenerative medicine and stem cells.

While this review focuses on tissue regeneration, AI can also play a vital role in medical planning. For instance, scheduling suitable appointment times can be challenging for some patients, but AI-driven self-care may offer solutions. Living with dental and oral problems often brings uncertainties. For example, a patient may have oral issues requiring examination but may be unsure if the problem can be resolved quickly. Additionally, predicting the causes of oral disease patterns can be challenging. Here, digital health tools and innovative technologies like AI can be instrumental. Developing software that analyzes oral cells in terms of structure and coverage can promote public health.

It is not just a theory as there are numerous widely used algorithms in dentistry today. For instance, Convolutional Neural Networks (CNNs) are widely applied in the diagnosis of dental radiographic images in order to diagnose caries, fractures or periodontal disease. They have enhanced the diagnostic accuracy through their ability to pick the smallest of patterns in imaging. Random Forests and Decision Trees can also be used in the predictive modeling and risk assessment of patients to forecast outcomes for oral conditions such as risk of oral cancer or effectiveness of a particular treatment. SVMs are important for classifying more intricate patient information, especially in the context of oral cancer to detect malignancies and better inform treatment. Used less frequently in dentistry than in general healthcare, NLP is used for extracting information from patients’ charts and clinical notes for data organization and use in individual treatment planning. ANNs are deployed in the predictive modeling of dental diseases to predict results and tailor patient care. In dental image classification KNN algorithms can be used, Fuzzy Logic can help to interpret the imprecise clinical data in decision-making, which will increase the accuracy of diagnostics [(Ammarullah et al., 2023; Hamet and Tremblay, 2017; Nsugbe, 2023)].

## 9 Limitations

Artificial intelligence (AI) has some major drawbacks when it comes to cell therapy, despite its potential advantages. The caliber and volume of data that is now available is one major constraint. For AI systems to forecast results with any degree of accuracy, they need large volumes of high-quality data. However, the data on stem cells in the field of cell therapy is frequently sparse and inconsistent, which makes it difficult to effectively train AI models. The quality of the training data has a direct bearing on how well AI predictions perform, and biases or inconsistencies in the data can have a big effect.

Moreover, the complexity of biological systems presents an additional challenge. The complex interactions between cells and tissues involved in cell therapy make it challenging for many machine learning and deep learning algorithms to simulate these processes precisely. A further limitation is the lack of focus on the sensitivity and specificity of AI compared to other common methods in most studies evaluating the effectiveness of AI. Except for hematopoietic stem cells, stem cell therapy is not currently FDA-approved and remains experimental, with an unclear understanding of its advantages and hazards. Considering this drawback, a precise definition of AI’s function in cell therapy may influence dental practice going forward. There are following challenges and limitations:

Data Availability and Quality: AI systems take a lot of high quality data to enable the system to run effectively. However, in the field of regenerative dentistry, information regarding the stem cell behavior, scaffold properties and patient report is generally scarce, contradictory or skewed. This limitation makes it difficult for the AI models to provide the right and quality predictions.
Complexity of Biological Systems: The dynamics of cell-tissue-scaffold systems are complex and cannot be easily simulated by today’s AI techniques. CNNs and ANNs, which are considered deep learning models, reveal high potential but they underestimate biological processes, which makes their predictions less accurate.
Sensitivity and Specificity: Most papers comparing the efficiency of AI in dentistry do not even mention that they have to define its sensitivity and specificity compared to standard methods. This lack of critical evaluation makes it difficult to build AI into an acceptable substitute or addition to the present methods.
Regulatory and Experimental Limitations: Many stem cell-based regenerative medicines are still clinical trials, and the FDA has not approved them. This regulatory limbo hinders the implementation of AI driven strategies into clinical scenarios.

## 10 Future directions

To fully realize the potential of AI in dental regeneration, several steps must be taken:

Development of High-Quality Datasets: The idea is to cooperate to obtain standardized and precise databases containing various patients and clinical environments. This would increase the chances of training and validation of AI models.
Integration of Multimodal Data: The current approach could be enhanced by the input of imaging, genomic data, and the patient’s records to enhance the accuracy of the AI and customise treatment plans.
Enhanced Algorithms: If these researchers were able to improve the algorithms that would map these biological interactions better, regenerative therapy with the help of artificial intelligence would be even more efficient. It is may be useful to integrate AI with the conventional biological systems if the models are hybrid in such cases.
Interdisciplinary Collaboration: Solutions to the problems encountered in the use of AI in dental practice require the input of dental practitioners, biomedical engineers, computer scientists together with ethicists.
Regulatory Frameworks: It is suggested that AI should be regulated in regenerative dentistry based on legislation of safety, responsibility and ethicality.

## 11 Conclusion

In general, artificial intelligence (AI) enables computers to think, reason, and solve problems in a manner akin to humans. Integrating dentistry with AI for the purpose of promoting regeneration and reconstruction may enhance treatment methods and reduce patient discomfort. The use of AI is constantly evolving and will significantly impact the future of the world. AI can track cell changes in real-time and has proven effective in evaluating the survival of dental pulp stem cells and investigating the differentiation of dental pulp cells. Moreover, AI can assist in the repair, reconstruction, or replacement of damaged or unhealthy tissues and organs using advanced approaches such as stem cell-based treatments, gene therapy, and tissue engineering.

The evaluation of oral tissue regeneration modeling using stem cells can aid in screening potential drugs and identifying alternative stem cells for further advancements in dentistry. This article, focusing on stem cells, provides insights into the role of AI in regenerative processes facilitated by stem cells.

AI has been successful in investigating the behavior of dental pulp stem cells. This technology can learn using human-like brain experiences and analysis, making it applicable to numerous medical decisions, including those in regenerative medicine. In the field of digital dentistry and cell therapy specifically, AI can be designed as a digital program or platform to identify patterns in cell type and morphological structure. In fact, AI examines all this data to identify cells with differentiated structures. The more data this technology processes, the more accurate its insights and recommendations become. Therefore, developing a tool with these characteristics could greatly benefit the healthcare sector.
